# Adolescents and parents’ perception of Young Person's Face IT: An online intervention for adolescents struggling with conditions affecting their appearance

**DOI:** 10.1177/20552076221147110

**Published:** 2022-12-25

**Authors:** Moa Millgård, Kristin B Feragen, Jeanette Ullmann Miller, Shahrzad Arfa, Heidi Williamson, Johanna Kling

**Affiliations:** 1 Centre for Rare Disorders, 155272 Oslo University Hospital, Oslo, Norway; 2 Centre for Appearance Research, 1981 University of the West of England, Bristol, UK

**Keywords:** Adolescent, parents, internet-based intervention, psychosocial intervention, body image, visible difference

## Abstract

**Objective:**

A visible difference in appearance caused by a congenital or acquired condition can negatively affect adolescents’ psychosocial well-being. Young Person's Face IT (YPF) is an online intervention based on cognitive behavioural therapy and social skills training, developed to help adolescents who struggle with adjusting to a visible difference. The objective of the present study was to explore adolescents’ and parents’ perceptions of the intervention's relevance and usefulness in supporting young people with appearance-related psychosocial concerns.

**Methods:**

Participants were adolescents (*N* = 76, aged 11–18) and parents (*N* = 15), recruited in a larger randomised controlled trial aiming at evaluating YPF. This qualitative study with descriptive data includes adolescents’ ratings on YPF's usefulness, and interview data from adolescents and parents on their experiences with YPF. The interviews were analysed using a thematic approach.

**Results:**

Results indicated that YPF was experienced as useful and relevant. Interviews showed that adolescents felt validated through the programme's content, discovered that other young people had similar experiences and felt that YPF could contribute to changing self-perceptions for the better. However, results could not confirm whether perceived usefulness led to the development and use of new social skills in real-life situations.

**Conclusion:**

This study offers new perspectives on the relevance and usefulness of YPF in supporting adolescents with appearance-related psychosocial concerns. Findings suggest that updates and modifications are required so that YPF stays relevant and useful for adolescents in need of support.

**Trial registration number:**

NCT03165331

## Introduction

Adolescents tend to be particularly concerned about their physical appearance, often compare themselves to others and want to fit in with their peers.^1^ Adolescence is also a period characterised by the formation of new peer relationships outside the family context and first experiences with romantic relationships.^2^ Experiences related to appearance and social interactions generally play an important role in the overall development of adolescents’ self-perceptions and psychosocial adjustment.^3^ Consequently, adolescence may be particularly challenging for young people with conditions that lead to an appearance appraised by society as undesirable and noticeably different to the norm (i.e. a visible difference).^4,5^

A visible difference can stem from scarring after injuries, burns or surgery, cancer treatment, skin conditions or congenital medical conditions that affect stature, limbs, face or skull.^5^ It can include a wide range of both rare and more common conditions, such as cleft lip and palate, Crouzon syndrome, psoriasis, acne, vitiligo, neurofibromatosis and alopecia.^6^

Research has shown that young people with a visible difference may be at greater risk of psychological and social difficulties than the general population, for example, in terms of social discrimination and mental health problems such as anxiety.^7–9^ However, adjustment is regarded as complex, highly variable and subject to individual differences. Evidence consistently shows that the association between the type, location and severity of a visible difference and psychological adjustment is low. Rather, psychological and social variables are believed to better predict distress.^10^ This includes the individual's subjective perception of how noticeable their difference is to others, and the extent to which they invest in and value appearance as an attribute contributing to their self-concept.^11^ Evidence also suggests that positive social experiences and supportive relationships with peers may serve as protective factors that facilitate adolescent adjustment to the challenges of looking noticeably different.^12^

Evidence that well-being is largely influenced by psychosocial factors, suggests that individuals may benefit from psychosocial interventions as an adjunct or alternative to medical intervention.^5^ However, access to specialised psychosocial support and treatment is limited,^13,14^ and there are few evidence-based age- and appearance-specific interventions available.^15^

To address these limitations, an online psychosocial intervention, Young Person's Face IT (YP Face IT; YPF), was developed by the Centre for Appearance Research (CAR), University of the West of England (UK), for young people aged 12–17.^14^ Co-produced with young people and specialist psychologists, the content is based on techniques from cognitive behavioural therapy (CBT) and social skills training (SST), and was informed by an adult version found to be successful at improving psychological functioning via a randomised controlled trial (RCT).^16^

YPF has seven weekly sessions, plus an additional booster session (a quiz) to maintain treatment effects that should be completed six weeks after Session 7. Participants complete the sessions independently. Sessions include multimedia interactive activities, testimonials and examples of real experiences shared by young people with a visible difference, and an audio recording as an alternative to text. Each session takes approximately 30–40 min to complete. The intervention is described in detail in Williamson et al.^17^

The feasibility and acceptability of YPF have been explored in several countries.^18–20^ In 2016, YPF was translated into Norwegian (Ung Face IT; www.ungfaceit.no)^21^ and the effectiveness of translated versions of YPF was recently evaluated in an RCT with participants from Norway and the Netherlands.^22^ The RCT showed that adolescents in the intervention group had significantly lower levels of social anxiety post-intervention compared with the control group (with a medium effect size).

Previous results in the Norwegian project showed that around 10% of participating adolescents displayed a clinically significant and reliable improvement in social anxiety and/or body esteem following YPF, and that a positive change was associated with more time spent on the programme and higher levels of distress at baseline. Based on the few previous quantitative evaluations of the Norwegian version of YPF, the intervention has displayed potential clinical effectiveness.^22,23^

However, gaining knowledge about participants’ perceptions of YPF, its acceptability, and their experiences of using it is also vital in order to inform its potential implementation. Two previous studies in the Netherlands and UK have qualitatively explored YPF participants’ experiences of the programme.^19,20^ Results indicated that the intervention generally was perceived as helpful and useful.^19,20^ Apart from not including sections on how to deal with social media, participants thought that the programme covered all relevant topics and issues.^19^ Opinions differed regarding the layout of the website as some commented that it was more appropriate for younger than for older adolescents.^19^ In addition, participants in the UK study described YPF as better suited to those with greater psychosocial concerns.^20^ In conclusion, results from previous qualitative evaluations of YPF indicate that the intervention is acceptable to Dutch and British adolescents. However, it should be noted that these two previous qualitative explorations were part of feasibility and acceptability studies and that the interviews largely focused on evaluating the research procedures (e.g. recruitment), rather than young people's in-depth experience of using the programme. In addition, it is important to consider the cultural sensitivity of the Norwegian translation of YPF when evaluating its acceptability and relevance.

### Aim

The aim of the present study was to strengthen the understanding of users’ experience with YPF. Specifically, we wanted to explore adolescents’ and parents’ perceptions of the intervention's relevance and usefulness in supporting young people with appearance-related psychosocial concerns in Norway.

## Method

### Study design

The present study was conducted at the Centre for Rare Disorders, Rikshospitalet, Oslo University Hospital, as part of a project evaluating the YPF intervention in Norway.^22^ The project was funded by the Research Council of Norway (trial registration number NCT03165331), reviewed by the Regional Committee for Medical Research Ethics (South-Eastern Norway Regional Health Authority, reference number: 2015/2440) and accepted by the Data Protection Office at Oslo University Hospital. All participants provided signed consent before enrolment. The present study was a qualitative study with descriptive statistical data, performed in two parts.

### Participants and procedure

#### Part 1. Descriptive statistical data

A total of 76 adolescents took part in the YPF intervention between June 2019 and September 2021. Eligibility screening was conducted via telephone by the research team using the following inclusion criteria: (a) age approximately 12–17, with self-identified psychosocial distress related to having a visible difference, (b) internet access and a home computer/tablet, (c) minimum reading level corresponding to that of a 12-year-old and (d) normal/corrected-to-normal vision. Exclusion criteria were: (a) diagnosis of psychosis, clinical depression, post-traumatic stress disorder (PTSD) and/or eating disorder, or within 12 months of traumatic injury, (b) learning disabilities that would hinder completion of the programme and (c) currently receiving professional psychological face-to-face support.

#### Part 2. Qualitative data: interviews

Adolescents involved in the intervention between October 2019 and August 2020 were invited (by telephone) to participate in the interviews and 25 accepted. The average interview time was 36 min with a range of 22–61 min. Parents of adolescents that took part in the YPF programme between October 2019 and August 2020 were also invited (by telephone) to participate in the interviews and 15 accepted. The mean average interview time was 34 min with a range of 18–56 min.

A research assistant and a doctoral student performed the interviews with the adolescents (*n* = 9 and *n* = 16, respectively) under supervision from KBF. The interviews with the parents were conducted by the research assistant (*n* = 1), the doctoral student (*n* = 11) and KBF (*n* = 3). All interviews were conducted via telephone to facilitate participation since participants were recruited nationwide.

#### Part 2. Qualitative data: interview structure

Based on the interview guide developed as part of the original UK YPF project,^17^ interviews with adolescents had two main parts: (a) exploring adolescents’ experiences with the YPF intervention and (b) exploring adolescents’ experiences of living with a visible difference. In line with our aim, the present study only included the first part of the interviews.

The interviews were semi-structured, with the following main questions: ‘What did you think of working through YPF?’, ‘What did you think of the tasks and activities within YPF?’ and ‘How does the programme reflect your own experiences of having a visible difference?’. Questions were followed up by a range of probes, for example, ‘What was good/not so good/difficult about it?’. Adolescents were also asked if they received support from others (i.e. ‘Did your parents or carers or friends help you with YPF at all?’).

The interview guide for the parent interviews (also based on the UK YPF project)^17^ had a similar two-part structure, but was shorter and focused on their child. The main questions in the parent interviews were: ‘What do you think your son/daughter thinks of YPF?’, ‘Did you assist your son/daughter in his/her work with the programme?’ and ‘Do you know if your son/daughter has used any of the advice or strategies from YPF?’ Probes, similar to those mentioned previously, also followed these questions. Importantly, parents did not have access to the YPF programme and had been instructed that adolescents generally were expected to complete the programme without active parental involvement. Parents were therefore not asked to comment on YPF's content or design.

Interviewers used the same interview guides, and no changes were made to the guides as data collection progressed.

### Data and data analysis

#### Descriptive data from the YPF programme

At the end of Sessions 1–6, adolescents had the option to rate four statements about the session on a scale from 1 (‘strongly agree’) to 5 (‘strongly disagree’). The statements were: (a) I thought the session was interesting, (b) I thought the session was easy to understand, (c) I thought the session helped me and (d) I thought the session could be improved. The original rating scale was constructed with a score of 3 to indicate uncertainty (‘Do not know’), making results from the scale statistically difficult to interpret. Therefore, we report results from the scale both with and without the ‘do not know’ option. The latter was done by recoding the scores 1, 2, 4 and 5 to 1–4, and reporting the number of ‘Do not know’ separately.

At the end of Session 7, adolescents were asked three questions about the programme as a whole (i.e. ‘How useful did you find the support?’, ‘Did you like taking part in the YPF programme?’, ‘Has YPF helped you change the way you do things?’) on a 10-point scale from ‘not at all’ to ‘very much’. They could also state their preferred mode of support (‘face-to-face’ or ‘YPF’). In addition, the present study included time usage per session per person (in minutes) and information about the number of sessions completed.

Descriptive analyses were performed using SPSS, version 28 (IBM Corp, Armonk, NY).

#### Interview data

All interviews were audio recorded and transcribed verbatim and analysed using a thematic, data-driven approach based on Braun and Clarke.^24,25^ Separate analyses were performed for the adolescents’ interviews and the parents’ interviews.

#### Analysis of adolescents’ interviews

The analysis included the following steps: MM and JUM independently coded five interviews. The coding process included a thorough and repeated reading of the transcripts, followed by writing descriptive words or phrases adjacent to associated excerpts. They then compared their findings, discussed and agreed upon the wording of codes and the inclusion of codes one or other had not identified. MM and JUM then coded the remaining interviews (*n* = 13 and *n* = 7, respectively).

Codes were grouped into themes and subthemes iteratively by MM who continuously compared back to the codes and excerpts. This thematic structure was reviewed and adjusted (i.e. subthemes were merged or separated) by MM, JK, KBF and JUM until all agreed on three overarching themes and five subthemes.

#### Analysis of parents’ interviews

The parents’ interviews were analysed separately from the adolescents’ interviews, by SA. To support an analysis process in line with the process for the adolescent's interviews, JK double-coded three interviews which were compared to SA's coding. After disagreements were discussed with KBF and resolved, SA linked the data elements together across the different codes and reorganised data extracts into two themes. These themes reflected (a) The usefulness of the programme and (b) Parents’ engagement in the programme. As the two themes identified in the parents’ interviews were considered by the research team to be fully captured by the themes and/or subthemes identified in the adolescents’ interviews, they were subsequently integrated with the adolescent data and are reported adjacent to the data from the adolescents’ interviews in the result section.

## Results

### Participant characteristics

Out of the 76 adolescent participants, 63% were girls. The mean age was 13.9 (*SD* = 1.73; range 11–18). All adolescents had a visible difference and experienced psychosocial challenges associated with their appearance (self-reported at enrolment in the project). Visible differences were due to a wide range of different craniofacial conditions (e.g. cleft lip and palate), skin conditions (e.g. eczema), related to body form (e.g. missing limbs) or due to scarring. The adolescents reported parental occupation/education: roughly 70% of the fathers and 90% of the mothers were in professions that require at least an undergraduate degree.

A sub-sample (*n* = 25) of the adolescents was interviewed (64% girls). The mean age in this subgroup was 14.0 (*SD* = 1.6; range 12–17). Out of these 25 adolescents, 24 had completed all seven sessions and 1 participant had completed four sessions at the time of the interview.

Out of the 15 participating parents (13 mothers), 6 were interviewed before their child had completed YPF.

### Descriptive statistical data

Table 1 provides details of adolescents’ usage and session feedback of YPF: 90% (*n* = 68) completed two sessions, 80% completed three and four sessions (*n* = 61), 71% five and six sessions (*n* = 54) and 53 adolescents completed all seven sessions (70%). The time adolescents spent on each session varied from 1 min (signed in but did not go through the session) to 128 min. Mean time per session ranged from 22 min (Session 7) to 47 min (Session 2).

**Table 1.** Number of adolescents completing sessions, time spent per session and assessment of session.

Session	*n* (%)	Minutes spent per session Mean (*SD*)	Minutes spent per session Median (Min–max)	*n* of responses (% of *n* completing session)	I thought the session was interesting	I thought the session was easy to understand	I thought the session helped me	I thought the session could be improved
Session 1	76 (100)	34.0 (22.6)	29.0 (1–89)	54 (71.1)	2 (1–5)	2 (1–5)	2 (1–5)	3 (1–5)
Session 2	68 (90)	47.0 (26.3)	45.5 (7–113)	53 (77.9)	1 (1–5)	1 (1–5)	2 (1–4)	3 (1–5)
Session 3	61 (80)	36.9 (23.1)	33.0 (3–97)	58 (95.1)	1 (1–4)	1.5 (1–4)	2 (1–4)	3 (1–5)
Session 4	61 (80)	35.3 (23.4)	29.0 (4–99)	50 (82.0)	2 (1–4)	1.5 (1–4)	2 (1–4)	3 (1–5)
Session 5	54 (71)	32.2 (28.6)	24.0 (3–128)	49 (90.7)	1 (1–3)	1 (1–4)	2 (1–4)	3 (1–5)
Session 6	54 (71)	25.6 (17.6)	23.0 (2–73.5)	46 (85.2)	1 (1–4)	1 (1–5)	2 (1–4)	3 (1–5)
Session 7	53 (70)	22.0 (17.8)	17.0 (2–88)	40 (75.5)	-	-	-	-
Booster session (quiz)	34 (45)	38.8 (21.8)	36.0 (1–79)	-	-	-	-	-

*Note*. Median (minimum to maximum range): 1 = strongly agree; 2 = agree; 3 = do not know; 4 = disagree; 5 = strongly disagree. The table is based on Williamson et al.^20^

Table 1 also details adolescents’ feedback on each session. Overall, median scores indicated a tendency for adolescents to rate the sessions as interesting, easy to understand and helpful. However, scores varied and ranged from minimum to maximum scores. A relatively high number of adolescents provided a score of 3 (‘Do not know’) when asked whether they experienced the sessions as interesting (*n* = 40), easy to understand (*n* = 37), helpful (*n* = 42) and whether sessions could be improved (*n* = 54). For the recoded variable (original score of 3 removed) and all sessions combined, median scores were: interesting (*Mdn* = 1, range 1–2; *n* = 24), easy to understand (*Mdn* = 1, range 1–2; *n* = 27), helpful (*Mdn* = 2, range 1–2; *n* = 22) and could be improved (*Mdn* = 3, range 2–4; *n* = 9).

Overall, adolescents (*n* = 29; 55% of adolescents completing all seven sessions) liked taking part in the programme (*M* = 8.07, *SD* = 2.07) and rated YPF as useful (*M* = 7.66, *SD* = 1.97). When asked whether YPF had helped them change the way they do things, mean scores were lower than for the two other items (*M* = 6.23, *SD* = 2.50; *n* = 30; 57%).

A total of 47 (89% of those completing YPF) answered a question about the preferred mode of support. Half preferred face-to-face support (*n* = 25; 53%), whereas the other half preferred YPF (*n* = 22; 47%).

### Qualitative results

Thematic analysis resulted in three main themes and subthemes (see Table 2). The first theme, ‘I am not the only one: validation of lived experiences’, depicts how YPF led adolescents to reflect on their visible difference. The second theme, ‘I have more courage to do things: coping and mastery’, describes possible changes in daily life after completion of YPF, whereas the third theme, ‘Some things were more useful than others: use of YPF’, includes participants’ thoughts regarding positive and negative aspects of YPF, its content and design. The main themes and subthemes are described in more detail and illustrated with participants’ quotes. For all themes, the adolescent perspective is presented first, followed by the parent perspective. However, since parents did not have systematic access to the programme, parental interview data are less comprehensive and not represented in all sub-themes.

**Table 2.** Overview of themes, sub-themes and example codes.

Main themes	Sub-themes	Example codes
1. I am not the only one: validation of lived experience	-	Social reactions Recognition
2. I have more courage to do things: coping and mastery	Increased awareness of thoughts and reactions Applying new skills in everyday life	Thoughts and emotions Use of strategies
3. Some things were more useful than others: use of YPF	Perceived value of YPF Potential pitfalls and challenges Parental support	Not relevant to me Time-consuming Support

### Theme 1. I am not the only one: validation of lived experiences

The first theme captures adolescents’ reflections on how YPF acknowledged their lived experiences and led them to reflect on their visible difference in a broader perspective.

Negative social reactions to their difference by others, such as staring or intrusive questioning, were recurring topics in most interviews with adolescent participants. Examples given by adolescents ranged from worrying and anticipatory thoughts about what other people might think or say, to experiences of teasing or bullying, and people talking behind their back. Adolescents also described how they tried to manage their reactions to these events and how YPF could or had helped them overcome the negative impact of social reactions to their visible difference. One participant described how he struggled with navigating between what he experienced as hurtful social reactions, while also trying to forgive and understand others' reactions.

When you get stared at for an eternity, and then they come up to you and like… well, they ask, (…) mostly young kids, (…) ‘what's that?’ (…). I just have to think: they’re just kids, they don't know any better. (…) I have experienced it a few times, but then it's been – yeah, for example, YPF has helped me. (Boy, aged 13)

Most adolescents saw similarities between the situations and examples described in YPF and their own experiences. One participant, in particular, described how he recognised situations, emotional reactions and avoidant behaviours presented within YPF.

That people looked at me… that I’ve been scared of going to parties because people would look at me in a funny way, or… that people on the bus stared at me, and then I felt weird, and… felt a bit different (…), I can really relate to that. (Boy, aged 14)

The importance of feeling represented and mirrored by testimonials provided within YPF was emphasised and described as comforting and empowering. When asked whether anything had changed for them after using the programme, a participant said:

Maybe a little. I felt pretty alone really… before. Now, I kind of know of others who are struggling too, with appearance, and maybe some who are a bit worse off too (…). Knowing this helps feeling less alone. You can actually remember that, you’re not alone then. (Girl, aged 17)

While most adolescents expressed that the content within YPF was relevant and recognisable, some adolescents felt that they related to given examples to a lesser extent, or not at all. Those who felt the content was less relevant typically had low levels of appearance-related distress or were confident in social settings. A few adolescents described how they found it helpful to compare themselves to those adolescents in YPF whose visible differences they perceived as more noticeable than their own.

I don't feel so special anymore, or like, weird, since there are lots of others who are struggling with a lot worse. (Boy, aged 14)

That's like ten times more visible than what I’ve got. So I feel a bit luckier than I initially thought. (Boy, aged 17)

In line with adolescents’ descriptions, parents expressed that YPF had helped their adolescent discover that other young people had similar experiences.

I think it has opened her eyes to the fact that there are others out there who may have the same problem as her, that she's not alone, and (…) that she can find answers in there [YPF programme], to questions we as parents perhaps don't have an answer to. (Father)

Some parents also noticed that their children had started to compare themselves to other adolescents with more severe conditions after participating in the programme, which helped them feel better about their own condition.

### Theme 2. I have more courage to do things: coping and mastery

The second theme illustrates possible changes to the adolescents’ self-perception and an increased sense of mastery and coping after using YPF. Parents seemed more hesitant about whether YPF had contributed to actual changes in daily life. This theme has two sub-themes: ‘Increased awareness of thoughts and reactions’ and ‘Applying new skills in everyday life’.

#### Sub-theme 1. Increased awareness of thoughts and reactions

Adolescents described ways in which YPF had changed how they thought about themselves and their visible difference. Some felt that YPF offered an opportunity to reflect on their habitual thought patterns, or helped them cope with overwhelming thoughts. One participant explained how this process had opened up possibilities to re-examine her beliefs and draw different conclusions.

I just started to think more about… all the thoughts that I have during the day. And which ones that might not be quite… quite true. (Girl, aged 16)

Some adolescents reflected on how their self-perception had changed for the better over the course of the programme. Although unable to express why, one participant stated a clear connection between a growing sense of pride and YPF.

I’ve become more proud of myself and stuff since I started with YPF. (Girl, aged 15)

Others shared how their fear of social interactions had decreased as their confidence in coping with social reactions had grown, with some describing a strengthened feeling of mastery over their thoughts and reactions.

I think I am managing [my situation] a lot better. (…) I used to be so scared, thinking I’m not going to get a boy/girlfriend, because I am so ugly. And no one is going to like me at the new school, for example. (…) But now, I’ve become more like, oh well, (…) if they think it's a bit weird, I can be friends with them anyway. (Boy, aged 14)

Parents generally expressed that YPF had strengthened their child's awareness of their own condition. Like the adolescents, some parents felt that working through the programme had provided a space for their children to reflect on their condition and the challenges they face on a daily basis.

…I have the impression that he has matured a bit through the project! … it kind of happened right after he had started with the programme, and I don't think that would have happened, if he had not taken part in YPF…. (Mother)

#### Sub-theme 2. Applying new skills in everyday life

Most adolescents shared examples of how they had tried techniques and strategies from YPF, although only some of them reported that they had used them in real-life settings. Others reported that they had not tried the techniques because they did not struggle with appearance concerns.

Adolescents specifically pointed out that examples and advice on how to respond to staring or questions about their visible difference (Session 1) were helpful, and had increased their confidence. One participant also found that using a stress-reducing exercise (Session 6) to help with social anxiety in a real-life situation had created hope for the future. It made her feel courageous and gave her a feeling of pride as she managed to overcome her fears.

I want to go to a concert, but (…) I’m really not sure if I want to, because of all the people that will be there. So I’m thinking that I can try, slowly but surely. (Girl, aged 15)

Another participant explained how YPF had helped her find alternative ways to prepare mentally for social interaction with strangers. While she could acknowledge her fear, she still managed to cope with it and could engage in a planned activity that was potentially challenging.

Instead of thinking ‘this is scary, I can't do it’, I can think ‘this is scary, but, what would happen if I do it? I will not see them again’. (Girl, aged 16)

Similarly, some parents shared that their children had learned strategies to overcome daily challenges. On the other hand, a few parents also pointed out that their children found it difficult to remember and/or use the techniques from the programme when facing real-life challenges. According to parents, a shift in behaviour in response to difficult situations was a demanding process for the child.

It's hard for him to change mindset, even if he, in a way, understands how it would be sensible to think or act, and he knows that the others probably don't think anything of it [the visible difference]… he has to go through it anyway… But he has reflected on the techniques…. (Father)

### Theme 3. Some things were more useful than others: use of YPF

The third theme captures the participants’ evaluation of the programme. This theme was categorised into three sub-themes, ‘Perceived value of YPF’, ‘Potential pitfalls and challenges’ and ‘Parental support’.

#### Sub-theme 1. Perceived value of YPF

This sub-theme describes participants’ views on the value of the programme, and whether they experienced the content as relevant.

Adolescents generally described YPF as comprehensive in terms of included themes and examples that reflected their lived experience. When asked, adolescents said that nothing was missing and they had no further examples of upsetting or uncomfortable experiences to add. The fact that the programme was self-administered and offered step-by-step methods was also described as helpful. When asked about what they thought of YPF, most adolescents expressed positive views, especially when talking about practical exercises and tips on how to handle specific challenging situations.

What I liked about it is that… it brings out things that might help, in a way. Not just like, if you’re being bullied (…), that's what happens. But it provides examples that may help as well, and there are exercises that may help to prevent it, so that you can ignore it the next time it happens to you. I think that's good. (Boy, aged 14)

Those who did not find the programme useful, nonetheless believed that YPF could probably benefit others*.* Two reasons were provided for not experiencing YPF as useful: (a) they experienced activities as less relevant when they did not feel that they suffered from appearance-related distress themselves and (b) that they had already gained the level of confidence needed to cope with social reactions to their visible difference. One participant, who had joined the study with the expectation that YPF also included strategies for weight-management, expressed a clear disappointment with YPF.

#### Sub-theme 2. Potential pitfalls and challenges

This sub-theme describes challenges or issues that limited the acceptability of YPF and that could reduce its beneficial outcomes.

Adolescents mostly pointed out that there was too much to read and/or that the programme was too time consuming. Some suggested that text-heavy sections could be replaced with videos. Long texts and extensive sub-sections felt wearisome to some adolescents, and led to them giving up and not completing a session, or not following up on suggested activities (‘homework’). The quiz in the booster session was typically described as helpful in repeating and helping them to recall what they had learned from YPF, although some adolescents thought it was too long and difficult.

Some adolescents found it hard to provide detailed and tangible recollections of how YPF had helped them and struggled with finding examples that could describe their perception of the programme,

I used much of it, but I can't remember any examples right now, if that makes sense? (Boy, aged 14)

Many adolescents also reported that they forgot to do the tasks and homework, or skipped parts of YPF.

In addition to broader questions about their thoughts on the programme, adolescents were also asked about specific features of the programme, such as the personal journal and the discussion forum. No adolescents had used the discussion forum and typically remarked that they had not even noticed its existence. Only some adolescents had used the journal, carefully noting their thoughts and experiences along the way, while others did not feel that using a journal was natural or useful for them.

Some adolescents believed that YPF was more suitable for a younger age group or perceived aspects of YPF as childish.

Maximum 15 [years of age] I guess. Because of the layout, and that's not the biggest issue, but some of the content is very basic stuff, most adolescents aged 17 already know. (Boy, aged 17)

Adolescents differed on how difficult they felt it was to understand the tasks and the content of YPF. Some considered it an easy programme, while others found it more challenging and struggled to understand messages within sessions. Other adolescents explained that it was complicated to understand and rate their own reactions and feelings.

I don't always know how I feel, which makes it difficult to answer (…) for example, when I’m asked to rate my anxiety. Like ‘When do you get most anxious?’, and then rate it from one to ten [becomes difficult]. (Girl, aged 12)

Some adolescents found parts of the programme to be emotionally and psychologically demanding, triggering negative emotional reactions, which made them initially hesitant to complete the programme. However, as pointed out by one of the adolescents, a challenging task did not necessarily lead to a negative experience.

‘I think it was really good. In the beginning, it wasn't easy, because I didn't want to do it, since I’m not a particularly open person when it comes to such things. (…) All of it wasn't fun, but eventually it went well, and the way you made [YPF] was really good. (Girl, aged 13)

Some parents were not sure how useful YPF was for their child, since they believed their child did not experience the type of problems described within YPF. Hence, some sessions were described as less relevant. As for the adolescents, they explained that YPF lacked relevance because of low levels of appearance-related distress.

#### Sub-theme 3. Parental support

Adolescents typically felt they did not need or want help to complete the programme. Other adolescents had support from their parents, for example, to understand specific words or questions. Some also expressed a need for emotional support, wished for their parents to be around while they worked through YPF, and believed that having their parent close by had contributed to the positive effects of YPF. For others, the programme offered an opportunity to have a conversation about visible difference as they talked to their parents about issues related to the programme.

I didn't need any help, but we talked about what I had gone through this week, and things like that. (Boy, aged 14)

When I read about for example how to deal with people who stare (…), they said, ‘we think you are pretty good at that’. And things like that. (Boy, aged 13)

Other adolescents shared that they had missed having the support of their parents while going through YPF.

I wanted someone to be there with me, to be able to talk to me in case something came up, and about what it said, so that they could help me with the answers if I wasn't sure, if I felt sad, and things like that. (Boy, aged 12)

Parents reported that generally they did not engage in their adolescent's work with YPF and explained that their child preferred and managed to use it on their own. Most adolescents did not share many experiences of YPF with their parents and some parents also said that their attempts to go through sessions together with their adolescent had been rejected. Parental involvement was mainly limited to reminding their adolescent to access YPF and complete each session, especially in the beginning. Finally, some parents expressed the wish to be more involved and suggested that information about each session and what their adolescent was expected to practice should be available to parents in advance.

Maybe we should somehow have had our own version, or a ‘shadow version’ of the programme… Maybe we should have had a little push to get involved. (Mother)

## Discussion

The aim of the present study was to explore adolescents’ and parents’ perceptions of YPF's relevance and usefulness in supporting young people with appearance-related psychosocial concerns in Norway. Results showed that most adolescents experienced YPF as useful and relevant, but it was not clear whether perceived usefulness had led to behavioural changes in real-life situations. Our findings are further elaborated.

### Contributions of YPF: validation and positive changes

Results suggest that YPF could be a useful resource for adolescents with a visible difference, and support parents and clinicians in their efforts to help this group of young people. Statistical data confirmed that most adolescents experienced YPF as helpful, interesting, and easy to understand, and median scores from the present study were very similar to those reported by Williamson et al.^20^ In addition, qualitative findings showed that many adolescents perceived the programme's content as validating and mirroring their lived experiences, which was described as comforting and empowering.

Other participants (adolescents and parents) felt that YPF did not meet their needs because the adolescent had lower levels of appearance-related concerns and perceived their visible difference to be less noticeable and stigmatising than examples provided within YPF. These findings, which align with previous qualitative^20^ and quantitative findings,^22,23^ reaffirm the value of obtaining subjective assessments of appearance concerns rather than relying on objective predictors of adjustment such as size and location of visible difference.

Through YPF, adolescents discovered that other young people live with a range of visible differences. This diminished feelings of loneliness and social difference and was considered supportive. Interestingly, several adolescents also mentioned that viewing other young people with more visible and stigmatising conditions had a positive effect, and in some cases, improved their self-perception. Parents also described that the adolescents’ comparisons with the young people in YPF that were considered to be ‘worse off’ (i.e. downward social comparison), made the adolescents feel better about themselves. This finding is in line with theories of social comparison,^26^ suggesting that people who compare with others who are thought to be faring worse, experience an improvement in their mood.

However, it should also be noted that social comparison could be problematic. For instance, previous body image studies have shown a clear association between social comparison and body dissatisfaction.^27^ Although this research mainly has focused on upward social comparison (e.g. comparing one's appearance with people considered more attractive), there are general adverse consequences associated with self-evaluation in relation to others. For instance, downward social comparison can help one feel better in the short term, but exacerbate negative self-perceptions in the long term.^26^ Therefore, we suggest that future studies could further explore the aspect of downward social comparison and its potential consequences in relation to YPF.

Adolescents who experienced YPF as useful also described how the programme had helped them prepare for challenging social interactions, provided them with more courage to try to handle difficult situations, and in some cases overcome social anxiety. Parents, however, voiced more scepticism regarding actual behavioural changes in real-life settings. Although adolescents and parents mentioned new strategies and techniques provided in YPF, adolescents struggled to provide examples of putting them into practice.

Nevertheless, YPF seemed to introduce a theoretical framework for behavioural change. Adolescents may need more support, time and encouragement to implement new techniques and skills in their everyday lives. Among those who experienced YPF as useful and relevant, the programme reportedly had the power to lead to positive changes in self-perceptions, enhanced feelings of coping and mastery and reduced loneliness and difference.

YPF includes seven sessions covering a range of different topics, and provides tools and tips aimed at enhancing understanding about visible differences in society, reducing social anxiety, strengthening social skills, social support networks, peer and romantic relationships. In the present study, adolescents particularly mentioned YPF's first session as appropriately representing the many social challenges related to unwanted social attention. The sixth session, that includes a stress-reducing exercise, was also specifically mentioned. Although the RCT study encouraged adolescents to complete all seven sessions, future users may benefit from more individual assessments and guidance on which sessions would be most applicable and relevant for them, as also mentioned in Riobueno-Naylor et al.'s study.^28^

### Considering criticised aspects of YPF

When adolescents were asked about negative aspects of YPF, they raised several specific issues. First, some felt there was too much to read, and one young person suggested replacing text with more videos. As a consequence, the programme was perceived as time-consuming, and some adolescents skipped parts of certain sessions or stopped going through YPF, as reflected by total time spent on sessions and intervention-adherence rates (Table 1). Nevertheless, other adolescents found YPF easy to complete and did not mention difficulties with progress or content.

Second, some adolescents mentioned that YPF could be perceived as somewhat childish. This was reported also in van Dalen et al.^29^ and Riobueno-Naylor et al.,^28^ who suggested tailoring the content to older adolescents. Based on our findings, developing a simpler and less time-consuming version of the programme, possibly for younger adolescents, should alternatively be considered.

Third, some adolescents mentioned that the design needed updating, which seems sensible given the fact that YPF was developed almost 10 years ago.^14^ Other features of YPF that were mentioned as possibly less useful, were the discussion forum and the journal, in line with previous pilot studies.^20,29^ For future implementations of YPF, a plan for how to continually update and improve the programme seems paramount to maintain its relevance and acceptability.

Fourth, some sessions were described as emotionally demanding by some, when information or activities triggered difficult feelings, such as anxiety or sorrow. A few adolescents had found a protective strategy for this potentially difficult situation; they ensured that their parents were available when going through YPF, so that they could ask for support if needed. However, older adolescents, or young people who struggle with sharing demanding emotions may not be able to use this strategy, and could potentially be among those not completing YPF.

Lastly, when asked about their preferred mode of support, 53% of the adolescents who completed the whole programme said they would prefer face-to-face therapy. It would be interesting for future research to explore whether adolescents who preferred YPF rather than face-to-face support were the ones reporting the most positive outcomes after using the programme.

### Parental support

YPF was developed as a self-help intervention, in line with many adolescents’ need to strengthen autonomy and build networks outside the parent and family context. However, while many adolescents managed to complete YPF on their own, some wished for or needed parental support. Parents also voiced that they had missed being more involved and informed. This result is in line with Riobueno-Naylor et al.,^28^ where parents missed having access to more parent-focused information on the content covered in YPF. The development of parental support versions that allow parents to learn and disseminate tools taught in YPF, at a pace they find appropriate for their own child, should possibly be a priority when working on implementation of YPF. Given some of the findings described above, future research should also examine whether parental involvement could have the potential to secure progression and completion of relevant sessions, provide needed emotional support, strengthen adolescents’ understanding of the programme's content, and increase the chances of practising new strategies and skills in real life settings.

Altogether, results point to the importance of gaining a better understanding of who benefits the most from YPF. Careful screening by a health care provider, such as a school nurse or counsellor, with the option of follow-up and regular support through the completion of the programme could be one way to achieve this. Active parental involvement may be the best option for some adolescents, while others may benefit from more autonomy while working through the programme. For this reason, tailoring supervision to individual needs may lead to better outcomes for the young person. Moreover, results from the present study confirm the centrality of building on the participatory principles that guided the development of YPF,^14^ ensuring participant feedback and involvement as key factors in the process.

### Limitations

The present study should be viewed in light of its limitations. The interview data were not particularly rich or elaborated. Parent interviews on the content of YPF were limited by the fact that most parents did not have access to the programme and at the start of the study were informed that it is a self-directed programme without active parental involvement. In addition, some were interviewed before their child had completed YPF. Consequently, many parents had little knowledge about the content of the YPF and their child's progress through the programme.

Interviews with the adolescents were also limited by two issues: (a) Two of the interviewers had little experience with qualitative interviews, and therefore possibly found it challenging to explore participants’ feedback with in-depth follow-up questions. Interviewing adolescents requires rapport building as well as careful consideration of the power dynamics between the young person and interviewer.^30^ The interviewer needs to make sure that the young person feels comfortable enough to engage in conversation and share their opinions. This lack of experience may have heightened young people's struggles with putting their thoughts into words and sharing them with the interviewers. (b) The main interviewer was also the first point of contact and technical support for the participants during the study. Therefore, it can not be ruled out that this relationship added a degree of social desirability to the adolescents’ responses, and that participants could have felt reluctant to share negative views on the programme. On the other hand, this relationship may also have provided participants with a sense of comfort during the interview, which could have been beneficial in building rapport with the interviewees.

Limitations also need to be mentioned regarding the descriptive statistical data. First, evaluation measures within YPF are not validated instruments. Second, in order for adolescents’ responses to register in YPF, adolescents had to press ‘save’ followed by ‘send’. This two-step procedure may have caused a loss of some responses. However, the response rate for those completing all seven sessions was still 75.5%.

It should also be noted that the majority of adolescents participated in the study during the COVID-19 pandemic. Social restrictions and lockdowns did not impede adolescents’ access to the programme, but may have limited their access to offline social environments and opportunities to practise learned skills and strategies during social interaction.

## Conclusions

The present study contributes with new perspectives on adolescents’ and parents’ perceptions of the online intervention YPF, and indicates that users perceive the programme as relevant and useful in supporting adolescents with appearance-related psychosocial concerns. Adolescents felt validated by the content of YPF and comforted by the revelation that many other young people live with similar experiences. Nevertheless, the current study and previous quantitative evaluations of the programme demonstrate that YPF is not suitable for all, and some adolescents may still need or prefer face-to-face interventions. Future research is needed to increase our understanding of whom benefits the most from access to YPF. Our findings also suggest that updates and modifications are required so that YPF stays relevant and useful for adolescents in need of support.
